# 1-[5-(Anthracen-9-yl)-3-phenyl-4,5-dihydro-1*H*-pyrazol-1-yl]ethanone

**DOI:** 10.1107/S160053681005018X

**Published:** 2010-12-11

**Authors:** Ming-Liang Wang, Bao-Li Dong, Yong-Hua Li

**Affiliations:** a School of Chemistry and Chemical Engineering, Southeast University, Nanjing 211189, People’s Republic of China

## Abstract

In the title compound, C_25_H_20_N_2_O, the pyrazoline ring is nearly planar [maximum atomic deviation = 0.0254 (17) Å]; but the anthracene ring system is distorted from a coplanar structure [maximum atomic deviation = 0.181 (3) Å], the dihedral angle between the outer benzene rings being 10.68 (13)°. The pyrazoline ring is almost perpendicular to the mean plane of the anthracene ring system [dihedral angle = 76.94 (8)°], but nearly coplanar with the phenyl ring [dihedral angle = 1.63 (7)°]. π–π stacking is observed between parallel benzene rings of adjacent anthracene units, the face-to-face distance being 3.27 (3) Å. Weak intra­molecular C—H⋯N hydrogen bonding also occurs.

## Related literature

For applications of pyrazoline derivatives, see: Christoph *et al.* (2003 ▶); Parmar *et al.* (1974 ▶); Soni *et al.* (1978 ▶); Wei *et al.* (2007 ▶). For a related structure, see: Krishna *et al.* (1999 ▶).
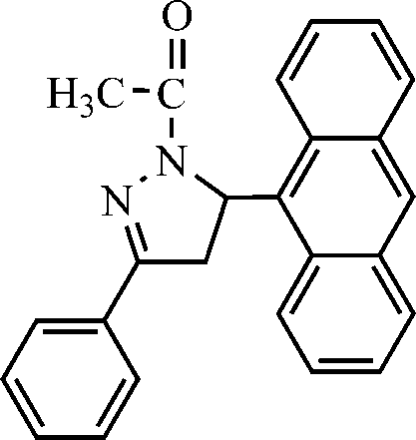


## Experimental

### 

#### Crystal data

C_25_H_20_N_2_O
*M* *_r_* = 364.43
Monoclinic, 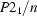

*a* = 8.7102 (17) Å
*b* = 16.251 (3) Å
*c* = 13.309 (3) Å
β = 91.49 (3)°
*V* = 1883.2 (7) Å^3^
*Z* = 4
Mo *K*α radiation
μ = 0.08 mm^−1^
*T* = 293 K
0.30 × 0.24 × 0.20 mm

#### Data collection

Rigaku SCXmini diffractometer
16656 measured reflections
3538 independent reflections
2021 reflections with *I* > 2σ(*I*)
*R*_int_ = 0.090

#### Refinement

*R*[*F* ^2^ > 2σ(*F* ^2^)] = 0.065
*wR*(*F* ^2^) = 0.142
*S* = 1.04
3538 reflections
255 parameters
H-atom parameters constrained
Δρ_max_ = 0.14 e Å^−3^
Δρ_min_ = −0.15 e Å^−3^

### 

Data collection: *CrystalClear* (Rigaku, 2005 ▶); cell refinement: *CrystalClear*; data reduction: *CrystalClear*; program(s) used to solve structure: *SHELXTL* (Sheldrick, 2008 ▶); program(s) used to refine structure: *SHELXTL*; molecular graphics: *SHELXTL*; software used to prepare material for publication: *SHELXTL*.

## Supplementary Material

Crystal structure: contains datablocks I, global. DOI: 10.1107/S160053681005018X/xu5095sup1.cif

Structure factors: contains datablocks I. DOI: 10.1107/S160053681005018X/xu5095Isup2.hkl

Additional supplementary materials:  crystallographic information; 3D view; checkCIF report

## Figures and Tables

**Table 1:** Hydrogen-bond geometry (Å, °)

*D*—H⋯*A*	*D*—H	H⋯*A*	*D*⋯*A*	*D*—H⋯*A*
C1—H1*A*⋯N2	0.93	2.55	3.404 (3)	152

**Fig. 1.**
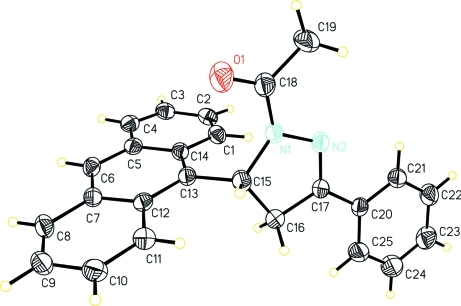
The structure of the title molecule. The displacement ellipsoids are drawn at the 30% probability level.

## supplementary crystallographic information

### Comment

Pyrazoline derivatives are important heterocyclic compounds and widely studied. Some of them have been used as pharmaceuticals for their broad spectrum of pharmacological activities such as antimicrobial, anticonvulsant, anti-inflammatory, analgesic [Parmar *et al.,* 1974, Soni *et al.,*1978]. Furthermore, some of them have widely been used as fluorescence probes in some elaborated chemosensors [Christoph *et al.*, 2003], as hole-transport materials in the electrophotography and electroluminescence [Wei *et al.*, 2007], due to the favorable photophysical properties. Here we report the structure of the title compound, a new derivative of pyrazoline.

In the pyrazoline ring, all the atoms are coplanar with a maximum deviation of 0.0254 (17)Å for atom C15, the bond length of N2=C17 [1.2878 (33)Å] agrees with normal C=N bond (1.28Å), the bond distance of N1-N2 [1.3905 (30)Å] conforms to the expected values [Krishna *et al.*, 1999]. The mean plane of pyrazoline ring makes dihedral angles of 1.63 (17)° and 76.94 (8)° with phenyl and anthryl ring, respectively. There are present only weak intermolecular interactions in the structure: C—H···π-electron and π-electron ring - π-electron ring interactions. The latter one is between the two parallel anthryl rings with the distance of 3.232Å. The anthryl ring shows a slightly distortion with C2 deviating by 1.811 (24)Å from planarity. The distance between the methine H15a and the anthryl H11a atoms is short, it is strange that the deviation of anthryl c11 from planarity is minimum in all the anthryl carbon atoms. It maybe result from the /p-stacking between the two parallel anthryl rings.

### Experimental

3-(9-Anthryl)-1-phenylprop-2-en-1-one (3 mmol) and hydrazine hydrate (50%, 6 mmol) were dissolved in 10 ml of glacial acetic acid. The mixture was stirred for 8 h at 391 K. The resultant solution was poured into a beaker containing crushed ice and the solid separated was collected by filtration. The product was recrystallized from ethanol-ethyl acetate (1:1 *v*/*v*) mixed solution, light yellow single-crystals of the title compound were obtained.

### Refinement

H atoms were positioned geometrically and refined using a riding model, with C—H = 0.93-0.97 Å, U_iso_(H) = 1.5U_eq_(C) for methyl H atoms and 1.2U_eq_(C) for the others.

### Crystal data

**Table:**

C_25_H_20_N_2_O	*F*(000) = 768
*M**_r_* = 364.43	*D*_x_ = 1.285 Mg m^−^^3^
Monoclinic, *P*2_1_/*n*	Mo *K*α radiation, λ = 0.71073 Å
Hall symbol: -P 2yn	Cell parameters from 3850 reflections
*a* = 8.7102 (17) Å	θ = 2.6–25.0°
*b* = 16.251 (3) Å	µ = 0.08 mm^−^^1^
*c* = 13.309 (3) Å	*T* = 293 K
β = 91.49 (3)°	Block, yellow
*V* = 1883.2 (7) Å^3^	0.30 × 0.24 × 0.20 mm
*Z* = 4	

### Data collection

**Table:**

Rigaku SCXmini diffractometer	2021 reflections with *I* > 2σ(*I*)
Radiation source: fine-focus sealed tube	*R*_int_ = 0.090
graphite	θ_max_ = 25.6°, θ_min_ = 3.0°
Detector resolution: 13.6 pixels mm^-1^	*h* = −10→10
φ and ω scans	*k* = −19→19
16656 measured reflections	*l* = −16→16
3538 independent reflections	

### Refinement

**Table:**

Refinement on *F*^2^	Secondary atom site location: difference Fourier map
Least-squares matrix: full	Hydrogen site location: inferred from neighbouring sites
*R*[*F*^2^ > 2σ(*F*^2^)] = 0.065	H-atom parameters constrained
*wR*(*F*^2^) = 0.142	*w* = 1/[σ^2^(*F*_o_^2^) + (0.0507*P*)^2^ + 0.2473*P*] where *P* = (*F*_o_^2^ + 2*F*_c_^2^)/3
*S* = 1.04	(Δ/σ)_max_ = 0.001
3538 reflections	Δρ_max_ = 0.14 e Å^−^^3^
255 parameters	Δρ_min_ = −0.15 e Å^−^^3^
0 restraints	Extinction correction: *SHELXTL* (Sheldrick, 2008), Fc^*^=kFc[1+0.001xFc^2^λ^3^/sin(2θ)]^-1/4^
Primary atom site location: structure-invariant direct methods	Extinction coefficient: 0.0174 (17)

### Special details

**Table:**

Geometry. All esds (except the esd in the dihedral angle between two l.s. planes) are estimated using the full covariance matrix. The cell esds are taken into account individually in the estimation of esds in distances, angles and torsion angles; correlations between esds in cell parameters are only used when they are defined by crystal symmetry. An approximate (isotropic) treatment of cell esds is used for estimating esds involving l.s. planes.
Refinement. Refinement of *F*^2^ against ALL reflections. The weighted *R*-factor *wR* and goodness of fit *S* are based on *F*^2^, conventional *R*-factors *R* are based on *F*, with *F* set to zero for negative *F*^2^. The threshold expression of *F*^2^ > σ(*F*^2^) is used only for calculating *R*-factors(gt) *etc*. and is not relevant to the choice of reflections for refinement. *R*-factors based on *F*^2^ are statistically about twice as large as those based on *F*, and *R*- factors based on ALL data will be even larger.

### Fractional atomic coordinates and isotropic or equivalent isotropic displacement parameters (Å^2^)

**Table:**

	*x*	*y*	*z*	*U*_iso_*/*U*_eq_	
N1	0.4492 (2)	0.69151 (12)	0.36622 (16)	0.0460 (6)	
N2	0.4001 (2)	0.61021 (12)	0.36062 (16)	0.0462 (6)	
O1	0.5886 (2)	0.79431 (13)	0.30449 (17)	0.0783 (7)	
C1	0.1365 (3)	0.76484 (17)	0.35515 (19)	0.0487 (7)	
H1A	0.1765	0.7119	0.3520	0.058*	
C2	0.0037 (3)	0.78223 (18)	0.30343 (19)	0.0529 (7)	
H2B	−0.0474	0.7406	0.2682	0.063*	
C3	−0.0573 (3)	0.86234 (18)	0.3024 (2)	0.0527 (7)	
H3A	−0.1465	0.8740	0.2653	0.063*	
C4	0.0144 (3)	0.92193 (17)	0.35558 (19)	0.0504 (7)	
H4A	−0.0245	0.9752	0.3528	0.061*	
C5	0.1481 (3)	0.90571 (15)	0.41604 (19)	0.0430 (7)	
C6	0.2101 (3)	0.96556 (16)	0.47959 (19)	0.0463 (7)	
H6A	0.1674	1.0180	0.4788	0.056*	
C7	0.3337 (3)	0.94940 (15)	0.54413 (19)	0.0441 (7)	
C8	0.3868 (3)	1.00942 (16)	0.6146 (2)	0.0521 (7)	
H8A	0.3380	1.0603	0.6168	0.063*	
C9	0.5058 (4)	0.99439 (18)	0.6782 (2)	0.0581 (8)	
H9A	0.5372	1.0339	0.7249	0.070*	
C10	0.5831 (3)	0.91788 (18)	0.67331 (19)	0.0579 (8)	
H10A	0.6663	0.9076	0.7167	0.069*	
C11	0.5375 (3)	0.85926 (17)	0.60629 (19)	0.0521 (7)	
H11A	0.5917	0.8100	0.6041	0.063*	
C12	0.4088 (3)	0.87087 (15)	0.53899 (18)	0.0402 (6)	
C13	0.3515 (3)	0.81000 (15)	0.47169 (18)	0.0416 (6)	
C14	0.2165 (3)	0.82539 (15)	0.41421 (18)	0.0404 (6)	
C15	0.4337 (3)	0.72775 (15)	0.46727 (19)	0.0485 (7)	
H15A	0.5368	0.7347	0.4972	0.058*	
C16	0.3539 (3)	0.65722 (15)	0.52291 (19)	0.0553 (8)	
H16A	0.4134	0.6405	0.5821	0.066*	
H16B	0.2518	0.6733	0.5429	0.066*	
C17	0.3464 (3)	0.58952 (15)	0.4462 (2)	0.0447 (7)	
C18	0.5338 (3)	0.72582 (18)	0.2927 (2)	0.0547 (8)	
C19	0.5526 (4)	0.67627 (19)	0.1987 (2)	0.0841 (11)	
H19A	0.5996	0.7097	0.1486	0.126*	
H19B	0.4538	0.6580	0.1742	0.126*	
H19C	0.6166	0.6294	0.2132	0.126*	
C20	0.2820 (3)	0.50776 (15)	0.4632 (2)	0.0456 (7)	
C21	0.2784 (3)	0.44894 (17)	0.3877 (2)	0.0548 (8)	
H21A	0.3193	0.4612	0.3256	0.066*	
C22	0.2152 (3)	0.37274 (17)	0.4034 (2)	0.0654 (9)	
H22A	0.2129	0.3343	0.3517	0.078*	
C23	0.1557 (4)	0.3528 (2)	0.4944 (3)	0.0726 (10)	
H23A	0.1140	0.3010	0.5048	0.087*	
C24	0.1582 (4)	0.4102 (2)	0.5699 (3)	0.0811 (10)	
H24A	0.1183	0.3971	0.6321	0.097*	
C25	0.2195 (4)	0.48683 (19)	0.5542 (2)	0.0690 (9)	
H25A	0.2190	0.5254	0.6057	0.083*	

### Atomic displacement parameters (Å^2^)

**Table:**

	*U*^11^	*U*^22^	*U*^33^	*U*^12^	*U*^13^	*U*^23^
N1	0.0528 (14)	0.0359 (13)	0.0500 (14)	−0.0003 (10)	0.0125 (11)	−0.0049 (10)
N2	0.0511 (14)	0.0351 (14)	0.0529 (15)	−0.0004 (10)	0.0099 (11)	−0.0014 (10)
O1	0.0781 (16)	0.0536 (14)	0.1048 (18)	−0.0203 (11)	0.0346 (13)	−0.0108 (12)
C1	0.0473 (18)	0.0493 (17)	0.0500 (17)	−0.0010 (13)	0.0088 (14)	−0.0074 (13)
C2	0.0492 (18)	0.064 (2)	0.0454 (17)	−0.0042 (14)	0.0061 (14)	−0.0068 (14)
C3	0.0457 (18)	0.065 (2)	0.0478 (17)	0.0071 (15)	0.0057 (13)	−0.0004 (14)
C4	0.0485 (18)	0.0535 (19)	0.0497 (17)	0.0095 (14)	0.0098 (14)	0.0053 (14)
C5	0.0421 (17)	0.0445 (17)	0.0430 (15)	0.0008 (12)	0.0136 (12)	0.0029 (12)
C6	0.0496 (18)	0.0370 (16)	0.0531 (18)	0.0044 (12)	0.0148 (14)	0.0007 (13)
C7	0.0500 (18)	0.0393 (16)	0.0438 (16)	−0.0041 (12)	0.0178 (13)	−0.0030 (12)
C8	0.059 (2)	0.0425 (17)	0.0554 (18)	−0.0078 (13)	0.0183 (16)	−0.0112 (13)
C9	0.073 (2)	0.055 (2)	0.0469 (18)	−0.0178 (16)	0.0110 (16)	−0.0100 (14)
C10	0.070 (2)	0.059 (2)	0.0444 (17)	−0.0115 (16)	−0.0028 (14)	0.0003 (14)
C11	0.061 (2)	0.0496 (18)	0.0457 (17)	−0.0015 (14)	0.0003 (14)	0.0004 (13)
C12	0.0431 (16)	0.0384 (16)	0.0394 (15)	−0.0048 (12)	0.0087 (12)	0.0042 (11)
C13	0.0469 (17)	0.0353 (15)	0.0433 (15)	−0.0005 (12)	0.0114 (13)	−0.0006 (11)
C14	0.0406 (16)	0.0378 (16)	0.0430 (15)	0.0002 (11)	0.0091 (12)	−0.0017 (11)
C15	0.0525 (17)	0.0430 (17)	0.0499 (17)	0.0043 (13)	−0.0007 (13)	−0.0057 (13)
C16	0.080 (2)	0.0434 (17)	0.0427 (16)	0.0065 (14)	0.0023 (14)	0.0010 (13)
C17	0.0510 (17)	0.0388 (17)	0.0443 (17)	0.0086 (12)	0.0014 (13)	0.0009 (12)
C18	0.0534 (18)	0.0440 (19)	0.068 (2)	−0.0033 (14)	0.0189 (15)	−0.0008 (15)
C19	0.112 (3)	0.068 (2)	0.075 (2)	−0.0151 (19)	0.053 (2)	−0.0067 (18)
C20	0.0470 (17)	0.0408 (17)	0.0494 (17)	0.0076 (12)	0.0051 (13)	0.0030 (13)
C21	0.0619 (19)	0.0480 (19)	0.0550 (19)	0.0023 (14)	0.0136 (14)	0.0019 (14)
C22	0.082 (2)	0.0430 (19)	0.072 (2)	−0.0049 (16)	0.0147 (17)	−0.0024 (15)
C23	0.080 (2)	0.053 (2)	0.086 (3)	−0.0091 (17)	0.0176 (19)	0.0107 (19)
C24	0.109 (3)	0.063 (2)	0.073 (2)	−0.009 (2)	0.032 (2)	0.0091 (19)
C25	0.098 (3)	0.055 (2)	0.055 (2)	−0.0081 (17)	0.0166 (17)	0.0012 (15)

### Geometric parameters (Å, °)

**Table:**

N1—C18	1.360 (3)	C11—C12	1.430 (3)
N1—N2	1.390 (3)	C11—H11A	0.9300
N1—C15	1.478 (3)	C12—C13	1.416 (3)
N2—C17	1.287 (3)	C13—C14	1.408 (3)
O1—C18	1.220 (3)	C13—C15	1.518 (3)
C1—C2	1.360 (4)	C15—C16	1.540 (4)
C1—C14	1.429 (3)	C15—H15A	0.9800
C1—H1A	0.9300	C16—C17	1.501 (3)
C2—C3	1.406 (4)	C16—H16A	0.9700
C2—H2B	0.9300	C16—H16B	0.9700
C3—C4	1.343 (4)	C17—C20	1.462 (3)
C3—H3A	0.9300	C18—C19	1.500 (4)
C4—C5	1.423 (4)	C19—H19A	0.9600
C4—H4A	0.9300	C19—H19B	0.9600
C5—C6	1.389 (3)	C19—H19C	0.9600
C5—C14	1.435 (3)	C20—C25	1.383 (4)
C6—C7	1.385 (3)	C20—C21	1.387 (3)
C6—H6A	0.9300	C21—C22	1.373 (4)
C7—C8	1.423 (3)	C21—H21A	0.9300
C7—C12	1.436 (3)	C22—C23	1.368 (4)
C8—C9	1.343 (4)	C22—H22A	0.9300
C8—H8A	0.9300	C23—C24	1.371 (4)
C9—C10	1.416 (4)	C23—H23A	0.9300
C9—H9A	0.9300	C24—C25	1.374 (4)
C10—C11	1.357 (4)	C24—H24A	0.9300
C10—H10A	0.9300	C25—H25A	0.9300
			
C18—N1—N2	121.5 (2)	C13—C14—C5	119.6 (2)
C18—N1—C15	123.8 (2)	C1—C14—C5	116.0 (2)
N2—N1—C15	113.1 (2)	N1—C15—C13	116.1 (2)
C17—N2—N1	108.6 (2)	N1—C15—C16	101.2 (2)
C2—C1—C14	122.1 (3)	C13—C15—C16	114.7 (2)
C2—C1—H1A	118.9	N1—C15—H15A	108.1
C14—C1—H1A	118.9	C13—C15—H15A	108.1
C1—C2—C3	120.9 (3)	C16—C15—H15A	108.1
C1—C2—H2B	119.6	C17—C16—C15	103.3 (2)
C3—C2—H2B	119.6	C17—C16—H16A	111.1
C4—C3—C2	119.5 (3)	C15—C16—H16A	111.1
C4—C3—H3A	120.2	C17—C16—H16B	111.1
C2—C3—H3A	120.2	C15—C16—H16B	111.1
C3—C4—C5	121.8 (3)	H16A—C16—H16B	109.1
C3—C4—H4A	119.1	N2—C17—C20	121.6 (2)
C5—C4—H4A	119.1	N2—C17—C16	113.6 (2)
C6—C5—C4	121.0 (2)	C20—C17—C16	124.8 (2)
C6—C5—C14	119.5 (2)	O1—C18—N1	120.0 (3)
C4—C5—C14	119.5 (2)	O1—C18—C19	123.1 (3)
C7—C6—C5	121.9 (2)	N1—C18—C19	116.9 (2)
C7—C6—H6A	119.0	C18—C19—H19A	109.5
C5—C6—H6A	119.0	C18—C19—H19B	109.5
C6—C7—C8	120.9 (2)	H19A—C19—H19B	109.5
C6—C7—C12	119.1 (2)	C18—C19—H19C	109.5
C8—C7—C12	120.0 (3)	H19A—C19—H19C	109.5
C9—C8—C7	121.5 (3)	H19B—C19—H19C	109.5
C9—C8—H8A	119.2	C25—C20—C21	117.6 (3)
C7—C8—H8A	119.2	C25—C20—C17	121.3 (2)
C8—C9—C10	119.4 (3)	C21—C20—C17	121.1 (2)
C8—C9—H9A	120.3	C22—C21—C20	120.8 (3)
C10—C9—H9A	120.3	C22—C21—H21A	119.6
C11—C10—C9	121.0 (3)	C20—C21—H21A	119.6
C11—C10—H10A	119.5	C23—C22—C21	120.7 (3)
C9—C10—H10A	119.5	C23—C22—H22A	119.6
C10—C11—C12	122.0 (3)	C21—C22—H22A	119.6
C10—C11—H11A	119.0	C22—C23—C24	119.3 (3)
C12—C11—H11A	119.0	C22—C23—H23A	120.3
C13—C12—C11	124.2 (2)	C24—C23—H23A	120.3
C13—C12—C7	119.8 (2)	C23—C24—C25	120.2 (3)
C11—C12—C7	116.0 (2)	C23—C24—H24A	119.9
C14—C13—C12	119.7 (2)	C25—C24—H24A	119.9
C14—C13—C15	121.5 (2)	C24—C25—C20	121.3 (3)
C12—C13—C15	118.7 (2)	C24—C25—H25A	119.3
C13—C14—C1	124.4 (2)	C20—C25—H25A	119.3

### Hydrogen-bond geometry (Å, °)

**Table:**

*D*—H···*A*	*D*—H	H···*A*	*D*···*A*	*D*—H···*A*
C1—H1A···N2	0.93	2.55	3.404 (3)	152
